# Direct observation of the effects of cellulose synthesis inhibitors using live cell imaging of Cellulose Synthase (CESA) in *Physcomitrella patens*

**DOI:** 10.1038/s41598-017-18994-4

**Published:** 2018-01-15

**Authors:** Mai L. Tran, Thomas W. McCarthy, Hao Sun, Shu-Zon Wu, Joanna H. Norris, Magdalena Bezanilla, Luis Vidali, Charles T. Anderson, Alison W. Roberts

**Affiliations:** 1 University of Rhode Island, 120 Flagg Road, Kingston, Rhode Island 02881 USA; 2 The Pennsylvania State University, 201 Huck Life Science Building, University Park, Pennsylvania, 16802 USA; 3 University of Massachusetts Amherst, 611 North Pleasant Street, Amherst, Massachusetts 01003 USA; 4 Worcester Polytechnic Institute, 60 Prescott Street, Worcester, Massachusetts 01605 USA; 5 Present Address: Indiana University, Bloomington, Indiana 47405 USA; 6 Present Address: Dartmouth College, Hanover, New Hampshire 03755 USA

## Abstract

Results from live cell imaging of fluorescently tagged Cellulose Synthase (CESA) proteins in Cellulose Synthesis Complexes (CSCs) have enhanced our understanding of cellulose biosynthesis, including the mechanisms of action of cellulose synthesis inhibitors. However, this method has been applied only in *Arabidopsis thaliana* and *Brachypodium distachyon* thus far. Results from freeze fracture electron microscopy of protonemal filaments of the moss *Funaria hygrometrica* indicate that a cellulose synthesis inhibitor, 2,6-dichlorobenzonitrile (DCB), fragments CSCs and clears them from the plasma membrane. This differs from Arabidopsis, in which DCB causes CSC accumulation in the plasma membrane and a different cellulose synthesis inhibitor, isoxaben, clears CSCs from the plasma membrane. In this study, live cell imaging of the moss *Physcomitrella patens* indicated that DCB and isoxaben have little effect on protonemal growth rates, and that only DCB causes tip rupture. Live cell imaging of mEGFP-PpCESA5 and mEGFP-PpCESA8 showed that DCB and isoxaben substantially reduced CSC movement, but had no measureable effect on CSC density in the plasma membrane. These results suggest that DCB and isoxaben have similar effects on CSC movement in *P. patens* and Arabidopsis, but have different effects on CSC intracellular trafficking, cell growth and cell integrity in these divergent plant lineages.

## Introduction

Cellulose is composed of β-1,4-glucan chains that are hydrogen-bonded together to form microfibrils, which are major contributors to the strength of plant cell walls. These microfibrils are synthesized by Cellulose Synthase (CESA) proteins that reside in the plasma membrane within Cellulose Synthase Complexes (CSCs). CSCs both polymerize β-1,4-glucan chains and facilitate their assembly into microfibrils. Mutations in Arabidopsis CESAs result in phenotypes that range from mild dwarfism to lethality, indicating the importance of cellulose in vascular plant development^1^. Much less is known about the function of cellulose in the development of non-vascular plants such as mosses^2^.

The study of CESAs and CSCs entered a new era with the development of methods for tagging CESAs with fluorescent proteins (FPs), facilitating live cell imaging of CSC movement behaviors^3^. These methods have facilitated investigations of CESA intracellular trafficking^4–7^, CSC interaction with the cytoskeleton and other proteins^8–11^, regulation of CESA and CSC function by endogenous and environmental factors^12^, and the mechanisms of action of cellulose synthesis inhibitors^13–18^, among other aspects of cellulose biosynthesis. All but one of these investigations have been performed in Arabidopsis, and imaging of CSCs in tip-growing cells has been precluded because FP-CESA fusion proteins fail to accumulate in the plasma membrane of these cell types^19^. Investigating cellulose synthesis in a nonvascular plant such as the moss *Physcomitrella patens* would enable us to better understand the evolution of cellulose synthesis and the functions of cellulose in a wider range of developmental processes, including tip growth.

The advantages of *P. patens* as an experimental organism include a high quality genome sequence^20,21^ and the capacity for targeted genetic manipulation due to its high rate of homologous recombination^22,23^. The *P. patens* plant body is typical of mosses, with two haploid stages: a filamentous protonemal stage, and gametophores consisting of leafy stalks with rhizoids^24^. The protonemal filaments extend by tip growth in a manner similar to the pollen tubes and root hairs of seed plant species^25–27^. The gametophore leaf cells expand by diffuse growth^28^ like most cell types in seed plants^29^.

Seven CESA isoforms have been identified in *P. patens*^30^. Mutation analysis has shown that *PpCESA5* is required for gametophore development^31^. *ppcesa5* knockout (KO) mutants have strong developmental phenotypes including failure of gametophore buds to sustain meristematic growth and produce leaves^31^. In addition, a subtle gametophore length phenotype has been reported for one double *ppcesa6/7* KO line^32^. We have recently found that *ppcesa8* KO mutants also have a developmental phenotype consisting of reduced cellulose deposition in the midrib stereid cells, which have thickened cell walls^33^. Because *ppcesa5* KO and *ppcesa8* KO lines have clear phenotypes, the functionality of mEGFP-PpCESA fusion proteins can be determined by testing transformed lines for complementation of these phenotypes.

One aspect of cellulose biosynthesis that has been clarified through the use of live cell CESA imaging is differences in the mechanisms of action between cellulose biosynthesis inhibitors^34^. In Arabidopsis, treatment with 2,6-dichlorobenzonitrile (DCB) immobilizes YFP-AtCESA6 in the plasma membrane, whereas treatment with isoxaben causes accumulation of YFP-AtCESA6 in vesicles below the membrane^14^. Although particle density was not measured, DCB reduced mEGFP-BdCESA particle velocity in *Brachypodium distachyon*^18^. In contrast, freeze fracture electron microscopy of protonemal filaments from the moss *Funaria hygrometrica* indicated that CSCs are lost from the plasma membrane after DCB treatment^35^. Freeze fracture examination of wheat roots treated for short periods with DCB showed increased CSC density in the plasma membrane of cortical cells^36^, indicating that this discrepancy is not due to differences in the CSC visualization method. DCB affects growth in widely divergent plants and related phyla, including red^37^, green^38^ and brown^39^ algae, but in most species little is known about its specific effect on CSCs. One possibility is that tip growing cells respond differently to DCB. The effects of DCB on pollen tubes of various plants such as lily, petunia^40^, and *Pinus bungeana*^41^ include distortion of cell walls and changes in cell wall composition^40,41^. Treatment with DCB also causes tip rupturing of pollen tubes^40^ and root hairs^19^, as well as moss protonemal filaments^35^. Treatment with isoxaben inhibits growth and induces tip swelling in conifer pollen tubes^42^ and retards growth in Arabidopsis root hairs^19^.

Here we show that CSC behavior can be analyzed by live cell imaging of mEGFP-PpCESAs in tip growing protonemal filaments of *P. patens*. Live cell imaging was applied to test the effects of the cellulose synthesis inhibitors DCB and isoxaben on both CSC behavior and protonemal filament growth. mEGFP-tagged PpCESA particles exhibited linear motility at the cell surface that was similar to the behavior of FP-CESA particles observed in Arabidopsis cells. Similar to results in Arabidopsis, treatment with DCB inhibited mEGFP-PpCESA particle motility without changing particle density at the cell surface. Whereas isoxaben treatment also greatly diminished mEGFP-PpCESA particle motility, it did not cause complete loss of particles from the cell surface in contrast to the case in Arabidopsis. Protonemal growth rates were not inhibited by either DCB or isoxaben treatment, but DCB treatment frequently resulted in tip bursting. Together, these data indicate that cellulose synthesis, cell growth control, and the regulation of wall integrity in tip growing protonemal filaments of *P. patens* share fundamental similarities and differences with these processes in diffusely growing Arabidopsis cells.

## Results

### Construction and characterization of mEGFP-PpCESA fusion protein expression lines

**Figure 1:**
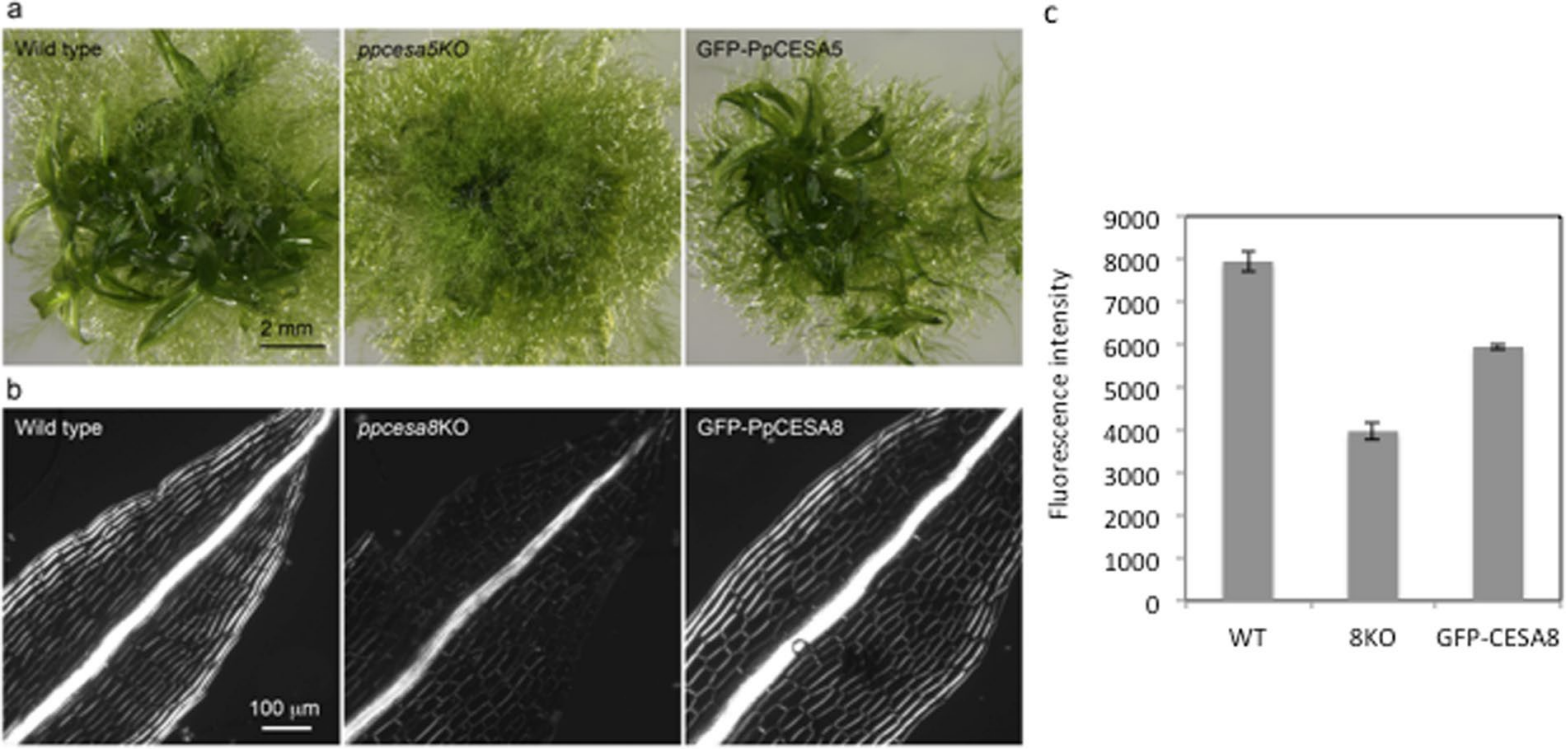
Expression of mEGFP-PpCESAs rescue the cognate mutant phenotypes. (**a**) Transformation of the gametophore defective *ppcesa5KO-2* line with *Ubi::GFP-PpCESA5* restores wild type gametophore development. (**b**) Transformation of the *ppcesa8KO-5B* line, which is characterized by weak midrib birefringence due to reduced secondary cell wall deposition, was partially restored to the wild type phenotype by transformation with *Act1::GFP-PpCESA8*. (**c**) Quantitative analysis of fluorescence intensity in midribs stained with S4B confirmed partial restoration of the wild type phenotype by expression of *Act1::GFP-PpCESA8* the *ppcesa8KO-5B* line.

To create FP-CESA fusion protein expression lines for live cell imaging of CESA dynamics, we transformed mEGFP-PpCESA expression vectors into cognate mutant lines with clear visual phenotypes. Transformation of *ppcesa5*KO-2^31^ with the *Ubi*::*mEGFP-PpCESA5* expression vector produced six stable hygromycin resistant lines in which the gametophore-deficient phenotype was rescued. We chose a single line for further analysis (Fig. 1a) based on screening for fluorescence intensity by spinning-disk confocal microscopy. Because our *ppcesa8*KO-5B line is hygromycin resistant^33^, we excised the *lox-p* flanked hygromycin resistance cassette by transient expression of the CRE protein^43^. Transformation of a hygromycin susceptible line with *Act1*::*mEGFP-PpCESA8* produced six stable hygromycin resistant lines. We chose one for further analysis based on screening for fluorescence intensity by spinning-disk confocal microscopy. Expression of mEGFP-PpCESA8 partially rescued the cellulose deficient midrib phenotype base on polarization microscopy (Fig. 1b). Quantitative analysis of S4B fluorescence intensity confirmed that the *Act1*::*mEGFP-PpCESA8* line is partially rescued (mean fluorescence intensity = 5,939 ± 146 (SE) Arbitrary Units (AU)) in comparison to the parental *ppcesa8KO* line (mean fluorescence intensity = 3,971 ± 247 (SE) AU) and the wild type (mean fluorescence intensity = 7,938 ± 247 (SE) AU). The mEGFP-PpCESA8 line differed significantly from both *ppcesa8KO* (p = 0.00101) and the wild type (p = 0.00101) based on the results of one-way ANOVA with Tukey HSD test. Although complementation was tested for gametophore phenotypes, both *PpCESA5* and *PpCESA8* are expressed in protonema^44^. We also tested for cellulose deficiency phenotypes in *ppcesa5*KO-2 and *ppcesa8*KO-5B (Fig. S1). No significant differences were detected. However, PpCESA3 and PpCESA8 are partially redundant in secondary cell wall deposition^33^ and constitutive expression of both PpCESA3 and PpCESA8 can rescue the *cesa5*KO gametophore phenotype (Scavuzzo-Duggan *et al*., in review). So, lack of a cellulose deficiency phenotype for the *cesa5*KO and *cesa8*KO protonema can be explained by functional redundancy, and thus does not preclude a role for PpCESA3 and PpCESA8 in normal protonemal cell wall deposition.

**Figure 2:**
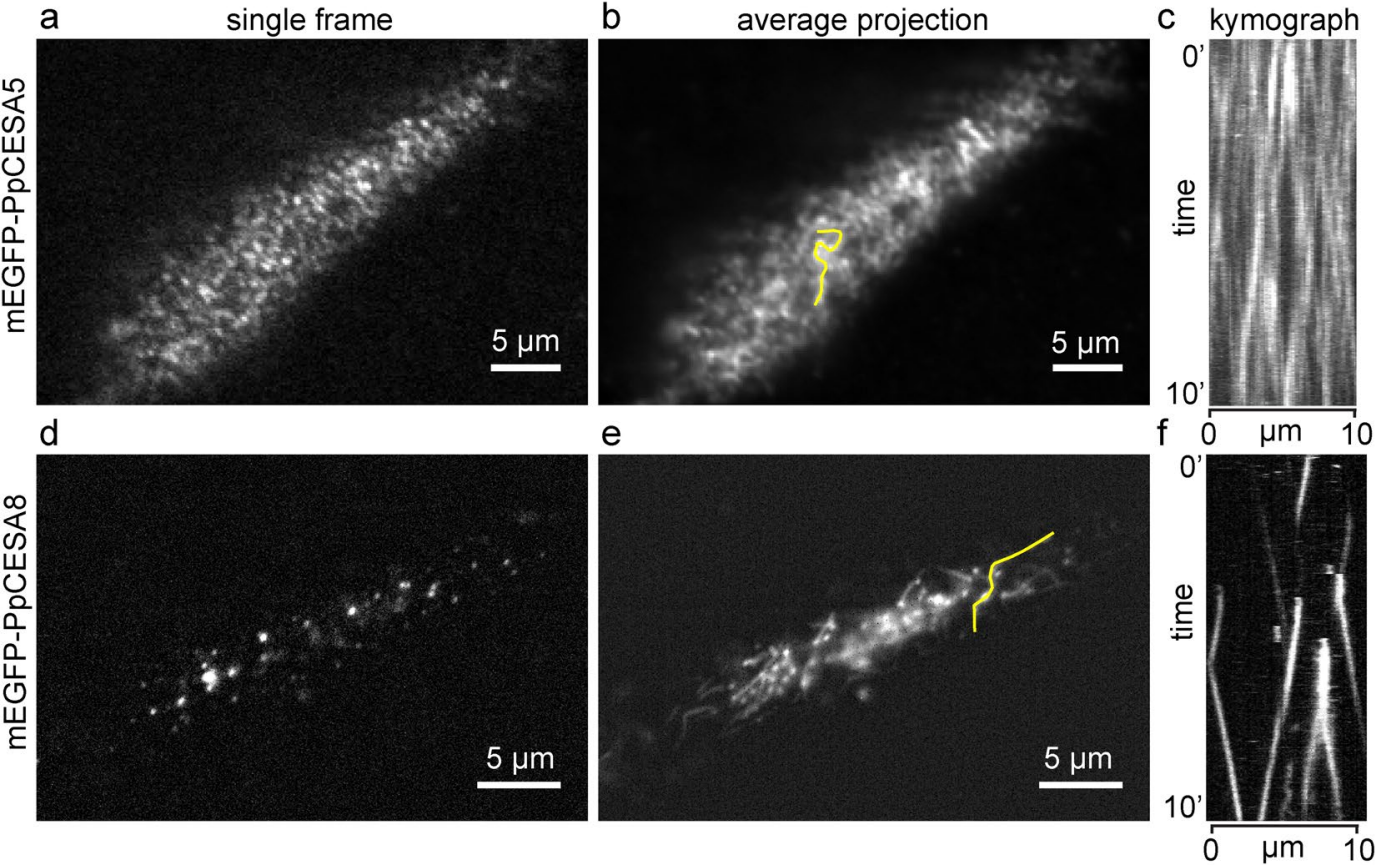
Imaging particle motility for mEGFP-PpCESA5 and mEGFP-PpCESA8. (**a**,**d**) Single frames from 10 min time-lapse imaging experiments. (**b**,**e**) Average projections of time-lapse images, with lines used to generate kymograph in yellow. (**c**,**f**) Kymographs of particle movement along lines in (**b**) and (**e**); diagonal lines represent movement over time. Images were acquired with VAEM with 2 s time interval.

After confirming that mEGFP-PpCESA5 and mEGFP-PpCESA8 rescue the phenotypes of *ppcesa5* and *ppcesa8* knockouts, respectively, we imaged these fusion proteins in protonemal cells using variable angle epifluorescence microscopy (VAEM). mEGFP-PpCESA5 particles were visible (Fig. 2a; Supplementary Movie S1), and moved across the cell surface along short linear paths (Fig. 2b,c) that were not aligned into co-linear arrays, as has been observed for FP-CESA particles that move in alignment with underlying cortical microtubule arrays in Arabidopsis^3^. We also detected mEGFP-PpCESA8 particles (Fig. 2d; Supplementary Movie S2), which moved across the cell surface along longer paths that were sometimes curved (Fig. 2e,f), but were also only rarely co-linear.

### Protonemal growth rate is not inhibited by DCB or isoxaben

**Figure 3:**
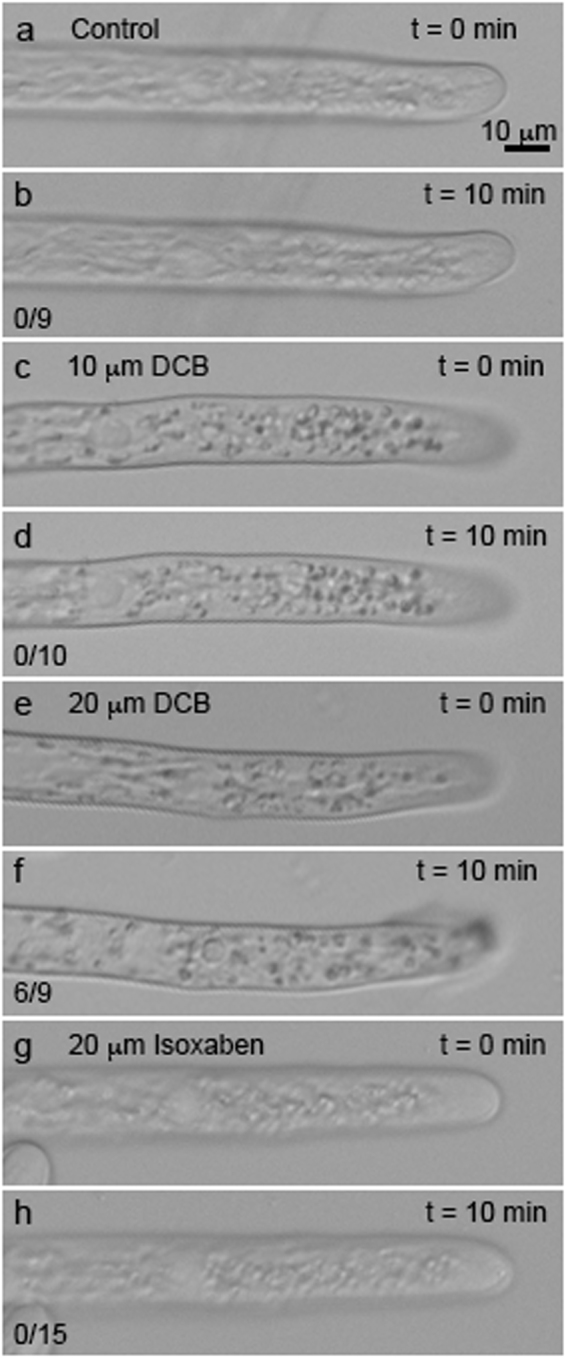
Protonemal tips rupture after treatment with DCB. Wild type *P. patens* protonemal filaments grown on solid PPNO_3_ medium for 7 days were treated with liquid PPNO_3_ medium with 0.05% ethanol (**a**,**b**), 10 µM DCB + 0.05% ethanol (**c**,**d**), 20 µM DCB + 0.05% ethanol (**e**,**f**), or 20 µM isoxaben + 0.05% ethanol (**g**,**h**) and were imaged at 30 sec intervals. Protonemal tips ruptured after treatment with 20 µM DCB (**f**), but not 20 µM isoxaben (**h**), or 10 µM DCB (**d**). The number of ruptured tips/number of imaged tips is shown for each treatment.

In preparation for testing the effect of cellulose synthesis inhibitors DCB and isoxaben on mEGFP-PpCESA particle movement, we examined their effect on *P. patens* protonemal filaments by live cell imaging to determine sensitivity relative to the related species *Funaria hygrometrica*^35^. In initial experiments, 6 of 9 protonemal tips treated with 20 µM DCB ruptured within 10 min. In contrast, none of the tips treated with 20 µM isoxaben (n = 15), 10 µM DCB (n = 10), or control medium containing 0.05% ethanol (n = 9) ruptured after treatment (Fig. 3; Supplementary Fig. S2). Even at isoxaben concentrations as high as 100 µM, no tip rupture was observed. To measure the effect of inhibitors on tip growth, we collected image series for 20 min (20 µM DCB) or 15 minutes (20 µM isoxaben and control) and subjected them to kymograph analysis (Supplementary Fig. S3). For tips treated with 20 µM DCB, 13 of 20 ruptured with a mean rupture time of 11.5 ± 1.1 (SE) min. Tip growth rates measured up to the time of rupture were 0.216 ± 0.0325 (SE) µm min^−1^. Tip growth rates for control and isoxaben treatments were 0.244 ± 0.0283 (SE) µm min^−1^ (n = 12) and 0.222 ± 0.0300 (SE) µm min^−1^ (n = 14), respectively, with no significant differences among any of the treatments (p = 0.832). Control and isoxaben treated tips did not rupture. These data indicated that cellulose synthesis inhibitors do not affect the kinetics of protonemal tip growth *per se* in *P. patens*, in agreement with data for *F. hygrometrica*^35^, but that DCB can affect protonemal cell integrity in actively growing cells.

### PpCESA-containing CSC particle motility, but not density at the cell surface, is reduced after DCB or isoxaben treatment

**Figure 4:**
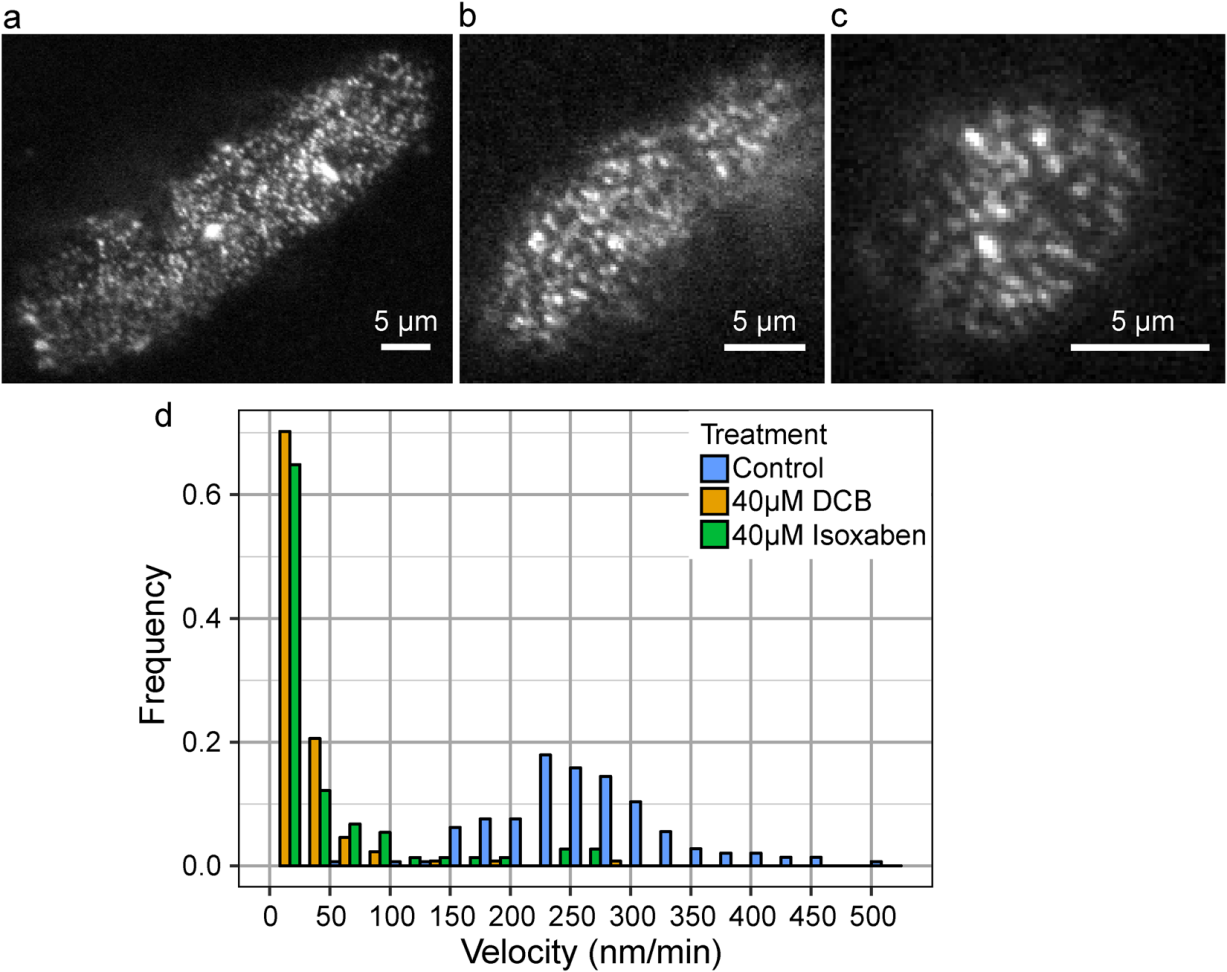
DCB and isoxaben inhibit mEGFP-PpCESA5 particle motility, but do not affect particle density at the cell surface. (**a**) Control protonemal cell treated with 0.1% ethanol. Mean CSC density = 0.48 ± 0.11 particles/μm^2^ (SD), n = 8 time-lapses. (**b**) Protonemal cell treated with 40 μM DCB. Mean CSC density = 0.51 ± 0.09 particles/μm^2^ (SD), n = 8 time-lapses. (**c**) Protonemal cell treated with 40 μM isoxaben. Mean CSC density = 0.44 ± 0.11 particles/μm^2^ (SD), n = 8 time-lapses. Scale bars = 5 μm. (**d**) Velocity distribution of mEGFP-PpCESA5 particles pooled from 24 time lapses. Mean velocities ± standard deviation in nm/min were: 262 ± 80 (Control); 18 ± 37 (40 μM DCB); and 36 ± 66 (40 μM isoxaben).

We then measured mEGFP-PpCESA particle density and motility under control conditions and after treatment with DCB or isoxaben. mEGFP-PpCESA5 particle velocities under control conditions averaged 262 ± 80 (SD) nm/min (Fig. 4a,d; Supplementary Movie S3), similar to the ~250–300 nm/min FP-CESA particle velocities measured in Arabidopsis seedlings^3,5,14^ and slightly faster than the mean of 164 nm/min measured in *B. distachyon* using a different quantification method^18^. Treatment of protonemal filaments expressing mEGFP-PpCESA5 with DCB or isoxaben before imaging dramatically decreased mEGFP-PpCESA5 motility (Fig. 4b–d; Supplementary Movies S4, S5). However, we did not measure large changes in mEGFP-PpCESA5 particle density at the cell surface after treatment with DCB and isoxaben, as compared to controls (Fig. 4a–c).

**Figure 5:**
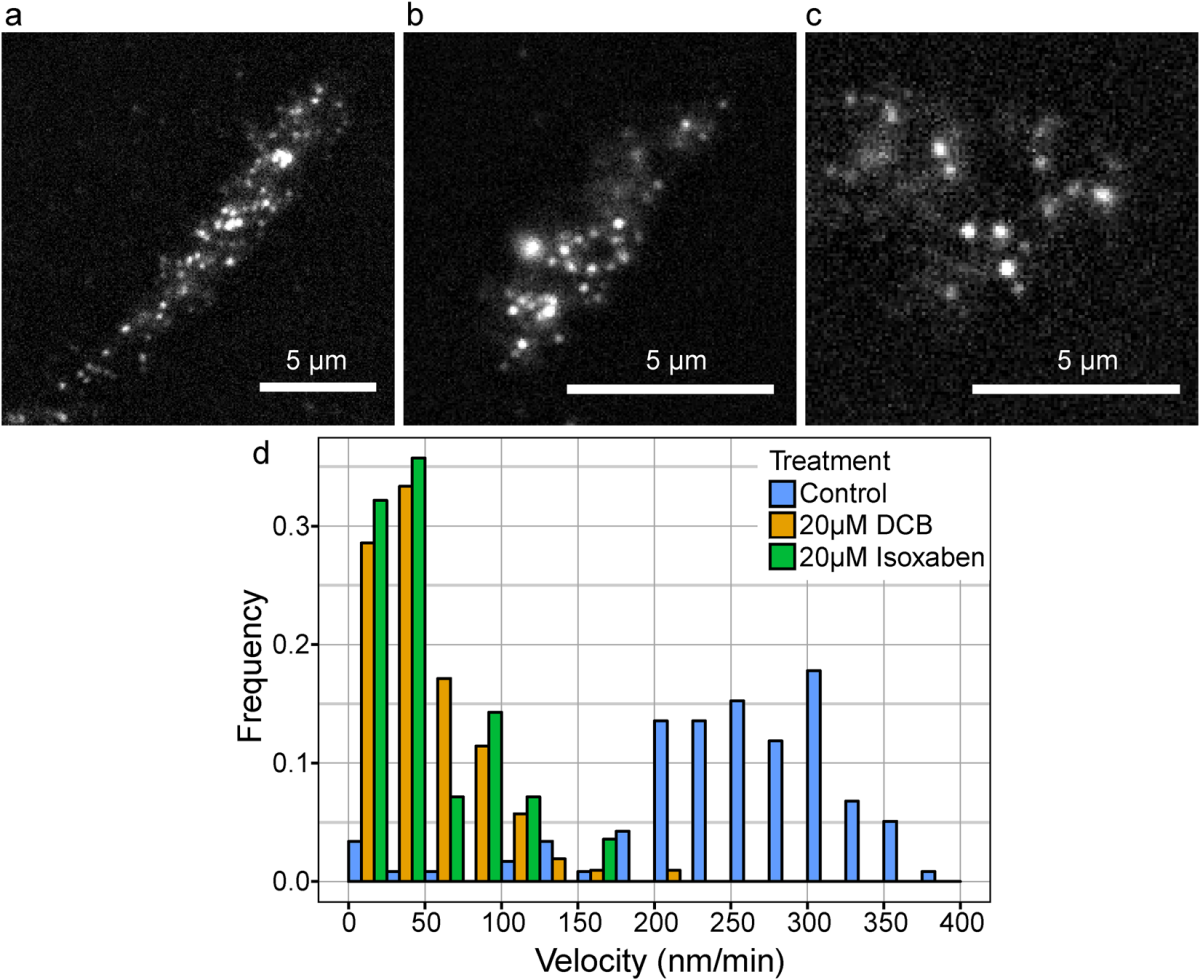
DCB and isoxaben inhibit mEGFP-PpCESA8 particle velocity, but do not affect particle density at the cell surface. (**a**) Control protonemal cell treated with 0.05% ethanol. Mean CSC density = 0.20 ± 0.09 (SD) particles/μm^2^, n = 11 time-lapses. (**b**) Protonemal cell treated with 20 μM DCB. Mean CSC density 0.15 ± 0.11 particles/μm^2^, n = 5 time-lapses. (**c**) Protonemal cell treated with 20 μM isoxaben. Mean CSC density 0.07 ± 0.09 (particles/μm^2^), n = 5 time-lapses. Scale bars = 5 μm. (**d**) Velocity distribution of mEGFP-PpCESA8 particles pooled from 21 time lapses. Mean velocities ± standard deviations in nm/min were: 253 ± 79 (Control); 47 ± 38 (20 μM DCB); and 42 ± 40 (20 μM isoxaben).

We also tested the effects of DCB and isoxaben on mEGFP-PpCESA8 particle velocity, which in control cells averaged 253 ± 79 (SD) nm/min (Fig. 5a,d; Supplementary Movie S6), remarkably similar to the velocities of particles containing mEGFP-PpCESA5. Also similar to the case for mEGFP-PpCESA5, treatment with DCB or isoxaben dramatically decreased mEGFP-PpCESA8 particle velocity compared to controls (Fig. 5b–d; Supplementary Movies S7, S8) with no large changes in mEGFP-PpCESA8 particle density at the cell surface. Although mEGFP-PpCESA8 particle densities were qualitatively lower than mEGFP-PpCESA5 particle density, we could not compare these densities statistically because they were estimated using different methods. In total, these results indicate that under control conditions, mEGFP-PpCESA particles behave similarly to FP-CESA particles in Arabidopsis in moving along linear trajectories at the cell surface at speeds of 250–300 nm/min, but that both DCB and isoxaben inhibit PpCESA particle motility and fail to completely remove FP-CESA particles from the cell surface, as has been reported for isoxaben-treated Arabidopsis cells^14^ .

## Discussion

Whereas live-cell CESA imaging in Arabidopsis has contributed greatly to our understanding of the cell biology of cellulose synthesis, AtCESA fusion proteins have not been observed in the plasma membrane in tip growing cells^19^. Using live-cell CESA imaging in *P. patens*, we have shown that PpCESA-containing particles move in the membranes of tip-growing cells with velocities similar to those observed in diffusely growing cells of Arabidopsis^3,5,14^. Complementation of *ppcesa* mutant phenotypes by expression of the cognate mEGP-PpCESAs provides support for the normal behavior of the fusion proteins expressed with either the rice *Actin1* promoter or maize *Ubiquitin* promoter.

The cellulose synthesis inhibitors isoxaben and DCB were confirmed to affect mEGFP-PpCESA5 and mEGFP-PpCESA8 motility, since velocities of PpCESAs decreased dramatically upon treatment with either cellulose synthesis inhibitor (Figs 4 and 5). The slowing of FP-PpCESA particle motility by DCB and isoxaben verify the potency of these drugs in *P. patens*, but their lack of effect on cell surface FP-PpCESA particle density and protonemal growth rates suggest that moss and seed plant cells respond to inhibition of cellulose synthesis by different mechanisms. In seed plants, cellulose synthesis inhibitors and *CESA* mutations activate a cell wall integrity-sensing pathway, resulting in production of reactive oxygen species, ectopic lignification, and growth inhibition (reviewed in^45^). It is possible that an inability to sense cell wall damage is responsible for the lack of growth inhibition following treatment with cellulose synthesis inhibitors in *P. patens*.

Although both DCB and isoxaben inhibited PpCESA motility, only DCB promoted bursting of protonemal tips. Differences in DCB and isoxaben mechanisms of action are manifested as opposing effects on CESA persistence in the plasma membrane in Arabidopsis^14^. Neither inhibitor altered CESA particle density at the cell surface in *P. patens*, in contrast to a report that the density of rosette CSCs as visualized by freeze fracture declined in response to DCB treatment in the related species *F. hygrometrica*^35^. In Arabidopsis, root hair tip growth is sensitive to DCB^46^, but not isoxaben^19^, perhaps because only DCB inhibits Cellulose Synthase-like D proteins (CSLDs), which contribute to cell wall deposition in root hairs and pollen tubes^19^. Thus, it is possible that CSLD activity is required to prevent bursting in both Arabidopsis root hairs and *P. patens* protonemal filaments.

Our data suggest that cellulose synthesis inhibitors affect CESA motility, and presumably the patterning and extent of cellulose polymerization, in *P. patens* protonemal cells, and also that tip growth in *P. patens* is differentially resilient to the inhibition of cellulose synthesis by these drugs. Differential effects, including the regulation of CSC trafficking and the sensing of cell wall integrity, will serve as fertile ground for future investigations into the unique and common mechanisms by which mosses and seed plants construct and expand their cell walls to control organismal growth and morphology during development.

## Materials and Methods

### *PpCESA* KO vector construction

Vectors for expressing mEGFP-PpCESA fusion proteins were constructed using Multisite Gateway Pro according to the manufacturer’s instruction (Invitrogen, Grand Island, NY, USA). A pDONR P1-P5r entry clone containing the coding sequence for mEGFP^47^ was linked to a pDONR P5-P2 entry clone containing the coding sequence of *PpCESA5*^48^ and inserted into the pTHUbiGate destination vector, which drives expression with the maize *Ubiquitin* promoter^49^. The P1-P5r mEGFP entry clone was linked to a pDONR P5-P2 entry clone containing the coding sequence of *PpCESA8*^33^ and inserted into the pTHAct1Gate destination vector, which drives expression with the rice *Actin1* promoter^50^. By using different destination vectors, we were able to test two constitutive promoters for use in this application. Both vectors confer hygromycin resistance and target the expression cassette to the intergenic 108 locus^22^. Vectors were cut with SwaI for transformation into their respective knockout lines.

### Culture and transformation of *ppcesa*KOs

All cultures were maintained in a growth chamber on basal medium supplemented with ammonium tartrate (BCDAT) as described previously^51^. The *hph* resistance cassette was removed from a *ppcesa8*KO-5B line^33^ by transforming protoplasts with NLS-Cre-Zeo, selecting for 7 d on BCDAT plates containing 50 μg mL^−1^ zeocin, replica plating the zeocin resistant colonies on BCDAT with and without 15 μg mL^−1^ hygromycin, and recovering hygromycin-sensitive colonies^43^.

*Ppcesa5*KO and *ppcesa8*KO-lox lines were transformed with mEGFP-PpCESA5 and mEGFP-PpCESA8 expression vectors, respectively, and subjected to two rounds of hygromycin selection as described previously^51^. Complementation of the *ppcesa5*KO^31^ and *ppcesa8*KO-lox^33^ morphological phenotypes was confirmed as described previously. Cellulose content of protonema cell walls was determined as described previously^33^ and the Kruskal-Wallis rank sum with Turkey-Kramer (Nemenyi) post hoc test (http://astatsa.com/KruskalWallisTest/) was used for statistical analysis.

GFP positive transgenic lines were selected using a Zeiss Cell Observer SD spinning disk confocal microscope (Zeiss, Carl-Zeiss-Strasse 22, 73447 Oberkochen) with a 488 nm excitation laser, a 525/50 emission filter, and a 100 × 1.40 NA oil immersion objective.

### Tip growth analysis by live cell imaging

For cellulose synthesis inhibitor assays, protonemal filament explants were cultured on PPNO_3_ solid medium^52^ in glass bottom petri dishes (P35G-0.17-14-C, Mat Tek, Ashland, MA) for 7 d under continuous light^53^. DCB and isoxaben (40 mM stock in 100% ethanol) were added to PPNO_3_ liquid medium^52^ at a final concentration of 10 or 20 μM (0.05% ethanol); 0.05% ethanol was added to control medium. The dishes were flooded with 100 μL of medium and filament tips were imaged every 30 sec for 20 to 25 min starting immediately after treatment using a Zeiss Axiovert 200 M DICII contrast microscopy (Zeiss) with dimensions set at 516 × 516 with AxioCam software. Image stacks were assembled into kymographs (Supplemenary Fig. S3) using the MultipleKymograph plugin in ImageJ (http://www.embl.de/eamnet/html/body_kymograph.html) and tip growth distance was calculated as described previously^53^. Means and standard errors were calculated from the combined results of two independent experiments. One-way ANOVA with post-hoc Tukey HSD test (http://astatsa.com/OneWay_Anova_with_TukeyHSD/) was used for statistical analysis.

### VAEM imaging of CESA-mEGFP particle trafficking

For VAEM imaging, 5- to 8-day-old plants regenerated from protoplasts were placed on an agar pad in Hoagland’s medium (4 mM KNO_3_, 2 mM KH_2_PO_4_, 1 mM Ca(NO_3_)_2_, 89 μM Fe citrate, 300 μM MgSO_4_, 9.93 μM H_3_BO_3_, 220 nM CuSO_4_, 1.966 μM MnCl_2_, 231 nM CoCl_2_, 191 nM ZnSO_4_, 169 nM KI, 103 nM Na_2_MoO_4_), covered by a glass cover slip and sealed with VALAP (1:1:1 parts of Vaseline, lanoline, and paraffin). For inhibitor treatments, 20 or 40 µM of DCB or isoxaben (40 mM stock in ethanol) was added to the Hoagland’s solution in the agar pad and imaging was started within 10 min. Controls were treated with the corresponding concentration of ethanol.

A Nikon Eclipse Ti microscope with a 100 X 1.49 NA TIRF objective and Andor DU-897 EMCCD camera was used to capture images every two seconds to create time-lapse videos of mEGFP-PpCESA5. A Nikon Eclipse Ti microscope with a 100X 1.49 NA TIRF objective and Zyla sCMOS camera (Zyla VSC-01746) was used to capture images every two seconds to create time-lapse videos of mEGFP-PpCESA8. Time lapses were from samples that were independently mounted and treated with inhibitor or solvent (5–8 time lapses per treatment).

### Analysis of CESA-mEGFP velocities

Time-lapse files were opened in Fiji image-processing software^54^. Image contrast was normalized to improve the visibility of particles. CSC velocity was measured by identifying each particle of a size and brightness consistent with Arabidopsis CSCs^3^ within the first slice of a time-lapse (time 0 seconds), measuring its position, tracking it until it was no longer visible, and measuring its last position. The displacement of the particle and the time necessary for travel were calculated to yield speed measurements for each particle. Particles that did not persist for at least one minute were excluded from analysis, as were particles whose size, speed, or brightness identified them as CESA-containing vesicles rather than CSCs. This process of identifying particles that appeared to be CSCs was repeated at slices corresponding to increments of 2.5 minutes, and identified particles were traced backwards and forwards through the time-lapse so that the beginning and end points would correspond to the first and last appearance of the identified particles. This was done to minimize bias in the experimenter toward particles that moved at “normal” CSC speeds.

### Analysis of mEGFP-PpCESA densities

To estimate CSC density in the mEGFP-PpCESA5 protonemal cells, time-lapse files were opened in Fiji and a Region of Interest (ROI) was selected with the freehand tool and its area measured with the measure function. The Particle Detector plugin was used to detect fluorescent CSCs with the following settings: 2 pixel radius, 0 cutoff, 1.9 percentile. The Particle Analysis Point Picker tool was used to select each particle within the ROI to acquire a count. Density was estimated as the number of particles detected by the Particle Detector plugin divided by the area of the ROI.

Signal levels of mEGFP-PpCESA8 particles were too low for the Particle Detector plugin to be able to reliably select CSC particles. Area of the cell was again determined using the freehand tool, but CSC count had to be approximated by the number of measured velocities from within the first slice of each time-lapse. This undercounted the number of CSCs because it ignored any that did not persist for 60s or whose paths could not be tracked during velocity measurements.

### Data availability

Additional images generated during the current study are available from the corresponding author on reasonable request.

## Electronic supplementary material

Supplementary Information

Supplementary Movie S1

Supplementary Movie S2

Supplementary Movie S3

Supplementary Movie S4

Supplementary Movie S5

Supplementary Movie S6

Supplementary Movie S7

Supplementary Movie S8
