# Supplementary material for: Direct observation of the effects of cellulose synthesis inhibitors using live cell imaging of Cellulose Synthase (CESA) in Physcomitrella patens
Source: Sci Rep. 2018 Jan 15;8:735. doi: 10.1038/s41598-017-18994-4 (PMC5768717; doi:10.1038/s41598-017-18994-4)
Supplement: Supplementary file 1 — Supplementary Information [file 41598_2017_18994_MOESM1_ESM.pdf]

Supplementary Information:

Direct observation of the effects of cellulose synthesis inhibitors using live cell imaging of Cellulose Synthase (CESA) in *Physcomitrella patens.*

Tran ML, McCarthy T, Sun H, Wu S-Z, Norris JH, Bezanilla M, Vidali L, Anderson CT, Roberts AW.

Supplementary Figures:

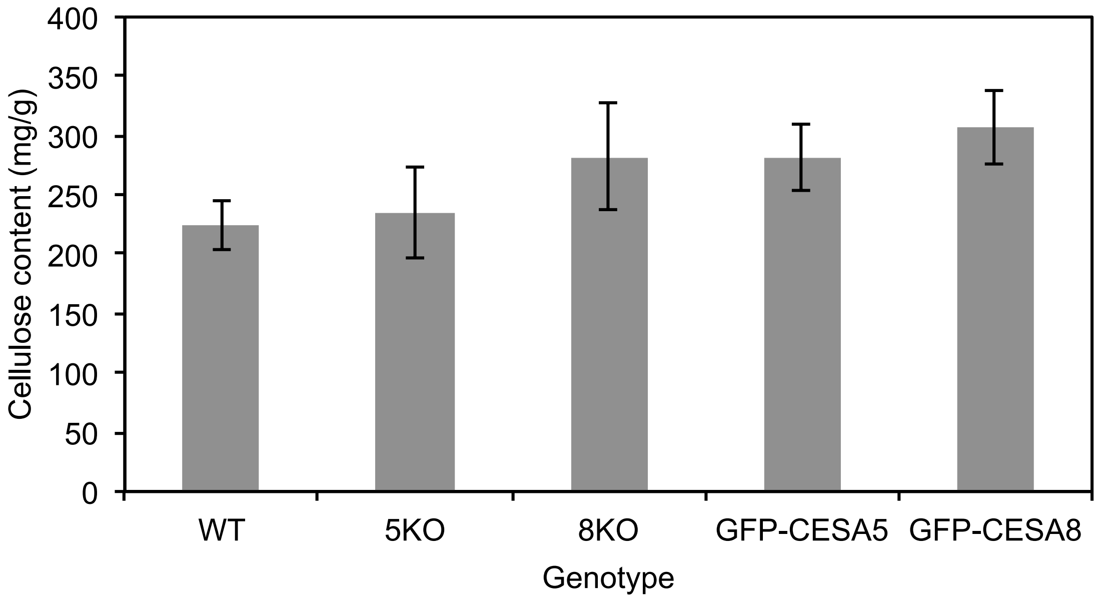

Fig. S1: Cellulose content of protonemal cells walls from wild type (WT), *ppcesa5*KO (5KO), *ppcesa8*KO (8KO), *Ubi*::*mEGFP-PpCESA5* (GFP-CESA5) and *Act1*::*mEGFP-PpCESA8* (GFP-CESA8) lines (3 technical X 3 biological replicates for each line). Error bars show standard deviations. Based on the Kruskal-Wallis rank sum with Turkey-Kramer (Nemenyi) post hoc test, all differences are insignificant with the exception of GFP-8KO compared to WT or 5KO.

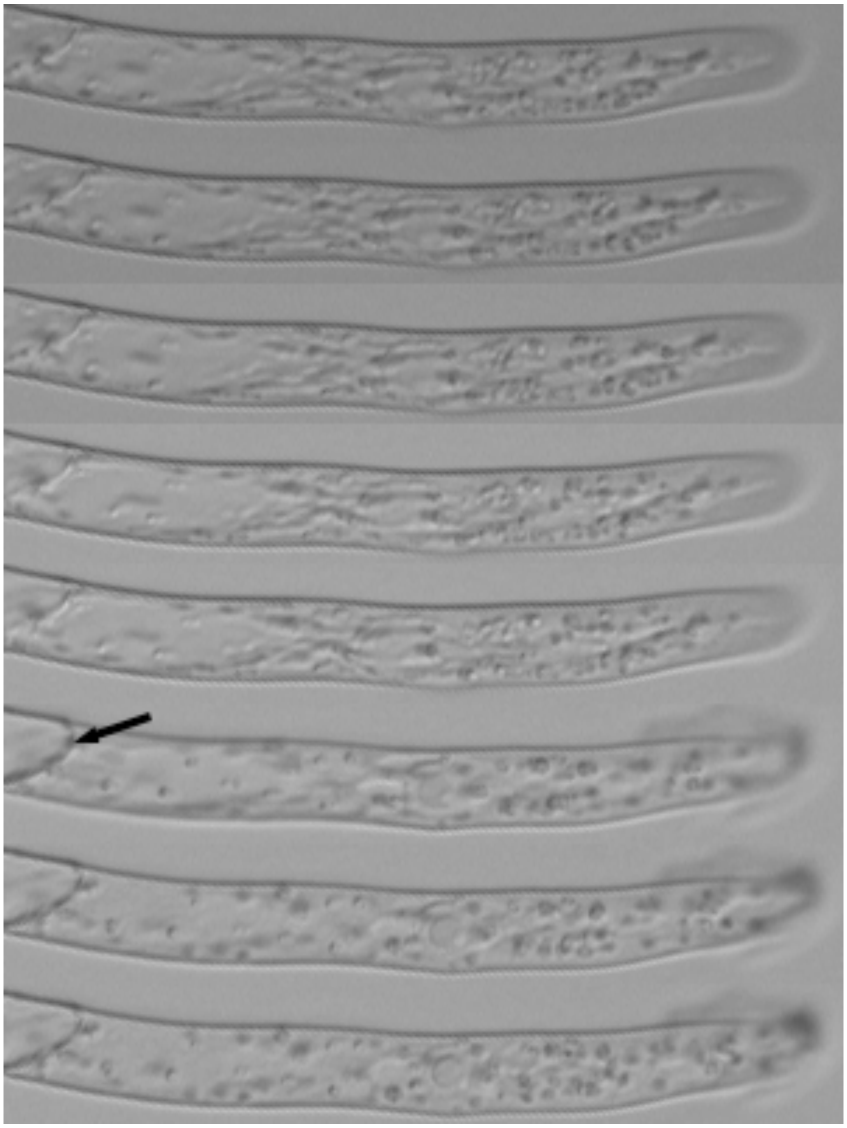

Fig. S2. Time lapse images of protonemal tip shown in Fig. 3e-f at 30 s intervals from t=6 min to t=9.5 min. Indications of tip rupture (t=8.5 min) include leakage of cytoplasm from the tip, loss of cytoplasmic strands, and bulging of the cross wall between apical cell and subapical cells (arrow).

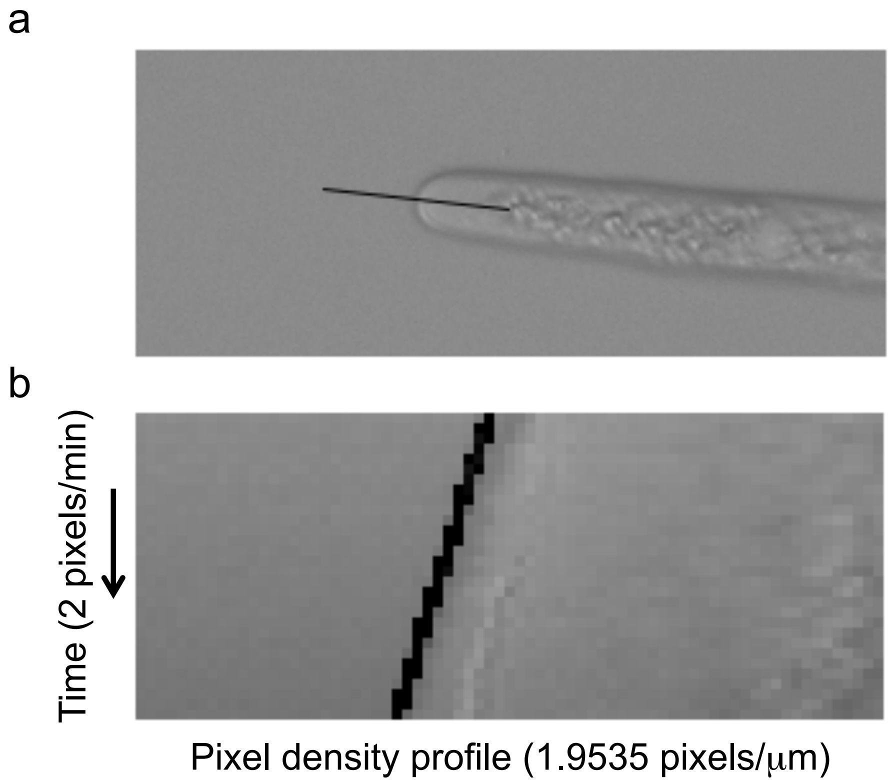

Fig. S3. Kymograph analysis. a) Line defined manually on the first image in a stack for generation of pixel density profiles. b) Kymograph of pixel density profiles from each image in the stack assembled top to bottom using the MultipleKymograph plugin in ImageJ (http://www.embl.de/eamnet/html/body_kymograph.html). Growth is calculated from the slope of a line fit to the slope of the signal (shown in black). Original resolution of the kymograph was 73x30 pixels.

Supplementary movie files:

Supplementary Movie S1: Movie of time-lapse corresponding to Figure 2a, mEGFP-PpCESA5.

Supplementary Movie S2: Movie of time-lapse corresponding to Figure 2d, mEGFP-PpCESA8.

Supplementary Movie S3: Movie of a representative time-lapse corresponding to Figure 4a, mEGFP-PpCESA5 control.

Supplementary Movie S4: Movie of a representative time-lapse corresponding to Figure 4b, mEGFP-PpCESA5 treated with DCB.

Supplementary Movie S5: Movie of a representative time-lapse corresponding to Figure 4c, mEGFP-PpCESA5 treated with isoxaben.

Supplementary Movie S6: Movie of a representative time-lapse corresponding to Figure 5a, mEGFP-PpCESA8 control.

Supplementary Movie S7: Movie of a representative time-lapse corresponding to Figure 5b, mEGFP-PpCESA8 treated with DCB.

Supplementary Movie S8: Movie of a representative time-lapse corresponding to Figure 5c, mEGFP-PpCESA8 treated with isoxaben.
